# Body mass index affects spirometry indices in patients with chronic obstructive pulmonary disease and asthma

**DOI:** 10.3389/fphys.2023.1132078

**Published:** 2023-12-01

**Authors:** Xiaohu Wang, Hui Gan, Yimin Wang, Xinxin Yu, Jiaying An, Baoqing Sun, Yi Gao, Zheng Zhu

**Affiliations:** ^1^ Department of Respiratory and Critical Care Medicine, People’s Hospital of Yangjiang, Yangjiang, China; ^2^ National Center for Respiratory Medicine, State Key Laboratory of Respiratory Disease, National Clinical Research Center for Respiratory Disease, Guangzhou Institute of Respiratory Health, First Affiliated Hospital of Guangzhou Medical University, Guangzhou, China; ^3^ Department of Allergy and Clinical Immunology, First Affiliated Hospital of Guangzhou Medical University, Guangzhou, China

**Keywords:** asthma, body mass index, chronic obstructive pulmonary disease, spirometry, obese

## Abstract

**Background:** Body mass index (BMI) is known to affect the outcomes of spirometry indices. However, its association with spirometry indices in COPD and asthma is less studied. We aimed to explore the impact of BMI on these patients.

**Methods:** Patients with COPD or asthma who completed bronchodilator tests (BDTs) between 2017 and 2021 were reviewed. Spirometry indices were compared among patients with COPD or asthma that were subclassified as underweight (BMI< 18.5 kg/m2), normal weight (≥18.5 to < 25), overweight (≥ 25 to < 30), and obesity (≥ 30). Results.

**Results:** Analysis was conducted on 3891 COPD patients (age:66.5 ± 7.8 years) and 1208 asthma patients (age:59.7 ± 7.5 years). COPD patients classified as underweight demonstrated significantly lower values of pre-and post FEV_1_ (L, %), pre-and post FVC (L, %), and pre- and post-FEV_1_/FVC (all *p* < 0.05). In contrast, COPD patients who were overweight or obese exhibited higher values for pre-and post FEV_1_ (L, %), and pre and post FEV_1_/FVC (all *p* < 0.05). Within the cohort of asthma patients, those underweight had lower pre-and post FEV_1_ (L, %), pre and post FVC (L, %), pre and post FEV_1_/FVC %. Obese asthma patients displayed higher pre and post FEV_1_/FVC (all *p* < 0.05).

**Conclusion:** Significant BMI category differences in spirometry indices can be seen in patients with COPD or asthma. Both underweight and obesity could affect the diagnosis and severity of these diseases. Recognizing these effects is essential to better management and diagnosis of these patients.

## Introduction

Spirometry plays a crucial role in the prognostication, diagnosis, and monitoring of chronic obstructive respiratory diseases, particularly chronic obstructive pulmonary disease (COPD) and asthma (Liou and Kanner, 2009; Halpin et al., 2021). International guidelines recommend the use of spirometry indices, specifically the pre- and post-bronchodilator test (BDT) forced expiratory volume in the first second (FEV1), forced vital capacity (FVC), and FEV1/FVC values, for the diagnosis and evaluation of COPD and asthma (Pellegrino et al., 2005; Halpin et al., 2021; Stanojevic et al., 2021; Reddel et al., 2022). It is important to note that these spirometry indices can be influenced not only by age, sex, and race, but also by factors such as height, weight, and body mass index (Talaminos Barroso et al., 2018; Mozun et al., 2022; Shah et al., 2022).

The impact of COPD and asthma on the global population is significant both financially and medically (GBD Chronic Respiratory Disease Collaborators, 2020). Lung function tests, particularly spirometry indices, should be correctly employed for the detection and treatment of these illnesses, as well as for understanding the factors that may affect them (Dharmage et al., 2023). BMI is a crucial factor associated with the nature and severity of COPD and asthma. Higher BMI has been linked to lower all-cause mortality and a decreased risk of COPD, but conversely, lower BMI has been linked to an increased risk of COPD and its exacerbations (Guo et al., 2016; Zhang et al., 2021; Shin et al., 2022). Furthermore, patients with asthma who have higher BMI also have higher respiratory burden scores (Klepaker et al., 2022). The effect of BMI categories on spirometry indices in COPD and asthma, however, has not been thoroughly researched. Therefore, investigations examining their connections are necessary.

In order to better manage, evaluate, and diagnose COPD and asthma, it may be valuable to recognize BMI differences in spirometry indices. Therefore, the aim of the present study was to explore the association of BMI with spirometry indices in COPD and asthma.

## Materials and methods

### Study design

This retrospective study included patients with COPD or asthma from the First Affiliated Hospital of Guangzhou Medical University who had at least one BDT between 2017 and 2021. The study was approved by the Ethics Committee of the First Affiliated Hospital of Guangzhou Medical University (approval NO.2020124). BMI was calculated based on the measurement of height and weight from the BDT record. BMI categories were classified into underweight (BMI< 18.5 kg/m^2^), normal weight (≥18.5 to <25), overweight (≥25 to <30), and obesity (≥30).

### Study population

Patients who underwent BDTs between 2017 and 2021 and had been diagnosed with COPD or asthma were included in the study. COPD was defined as a post-BDT FEV_1_/FVC ratio less than 0.7 according to the Global Initiative for Chronic Obstructive Lung Disease guidelines (Halpin et al., 2021). Asthma was defined on the basis of a history of asthma diagnosis by a pulmonologist that met the diagnostic criteria of the Global Initiative for Asthma guidelines (Reddel et al., 2022). Exclusion criteria: 1) Quality control of pulmonary function testing was not acceptable; 2) Patients who had other chronic respiratory diseases, such as lung cancer, bronchiectasis, interstitial lung disease; 3) The weight or height value was incomplete.

### Bronchodilator tests

BDTs were performed using the MasterScreen-Pneumo PC (Jaeger, Germany) and QuarkPFT (COSMED, Italy) spirometers (Wang et al., 2021). All test procedures and results interpretation were performed by trained technicians based on the American Thoracic Society and European Respiratory Society guidelines (Pellegrino et al., 2005; Graham et al., 2019). At least three acceptable maneuvers were needed and the highest measurement is used. Spirometry parameters including FEV_1_, FVC, and FEV_1_/FVC were obtained and expressed as absolute and %predicted (%) values. Reference equations for predicted values of spirometry indices were using the Global Lung Function 2012 equations (Quanjer et al., 2012).

### Statistical analysis

Data are presented as absolute numbers (percentages) in case of frequencies, median values and quartiles in case of continuous parameters. Count data were analyzed by using the χ2 test. Median inter-group comparisons were using the Kruskal–Wallis test. A *p*-value of <0.05 was considered statistically significant. Statistical analyses were performed with SPSS version 26.0.

## Results

### Prevalence of BMI categories

A total of 3,891 (93.0% male, n = 3619) patients with COPD were included, with a mean BMI of 21.9 ± 3.5 kg/m^2^, age 66.5 ± 7.8 years. Among them, there were 675 (17.3%) underweight, 2,496 (64.1%) normal weight, 646 (16.6%) overweight, and 74 (1.9%) obesity. There were 1,207 patients with asthma included, mean BMI 23.9 ± 3.3 kg/m^2^, age 59.7 ± 7.5 years. Among them, there were 63 (5.2%) underweight, 707 (58.5%) normal weight, 393 (32.5%) overweight, and 45 (3.7%) obesity.

### Demographic and clinical characteristics

Table 1 demonstrates the demographic and clinical characteristics of patients with COPD. Patients in the underweight group were more likely to be older compared to the overweight group (*p* = 0.007). In addition, the obesity group showed a lower ratio of female patients in comparison with the underweight and overweight groups (*p* = 0.014). There were no significant differences among all groups in smoking status and symptoms including cough, sputum, and shortness of breath.

**TABLE 1:** Demographic and clinical characteristics of patients with COPD.

Parameters	BMI categories				*p*-Value
	Normal weight (≥18.5 to <25) N = 2,496	Underweight (<18.5) N = 675	Overweight (≥25 to <30) N = 646	Obesity (≥30) N = 74	
Age, year	66 (61, 72)	67 (62, 72)^a^	65 (60, 71)^b^	66 (62, 70)	**.007** ^c^
Sex, male/female	2,327/169^d^	636/39^d^	592/54	63/11^b^ ^,^ ^e^	**.014** ^f^
Smoking status, n (%)					.496^f^
Current/former smoker	679 (27.2)	196 (29.0)	183 (28.3)	16 (21.6)	
Never smoker	1,817 (72.8)	479 (71.0)	463 (71.7)	58 (78.4)	
Symptoms, n (%)					
Cough	1,561 (62.5)	407 (60.3)	400 (61.9)	40 (54.1)	.376^f^
Sputum	1,694 (67.9)	469 (69.5)	425 (65.8)	43 (58.1)	.159^f^
Shortness of breath	1,559 (62.5)	426 (63.1)	393 (60.8)	43 (58.1)	.718^f^

Data are presented as absolute numbers (percentages) in case of frequencies, median values and quartiles in case of continuous parameters.

^a^ *p* < 0.05, overweight group versus the remaining groups;

^b^ *p* < 0.05, underweight group versus the remaining groups;

^c^ *p*-value is calculated using Kruskal–Wallis tests.

^d^ *p* < 0.05, obesity group versus the remaining groups.

^e^ *p* < 0.05, normal weight group versus the remaining groups;

^f^ *p*-value is calculated using χ2 test.

Significant *p*-values indicated in bold text. BMI, body mass index.

In asthma, patients in the underweight group were older than normal weight and overweight groups (*p* = 0.002). The obesity group had a lower ratio of female patients when compared with the normal and overweight groups. Same as patients with COPD, patients with asthma showed no significant differences among all groups in smoking status and symptoms. Detailed information can be found in Table 2.

**TABLE 2:** Demographic and clinical data of patients with asthma.

Parameters	Normal weight (≥18.5 to <25) N = 707	Underweight (<18.5) N = 63	Overweight (≥25 to <30) N = 393	Obesity (≥30) N = 45	*p*-Value
Age, year	58 (54, 64)^a^	63 (56, 66)^b^ ^,^ ^c^	58 (53, 64)^a^	61 (56, 65)	.002*
Sex, male/female	329/378^d^	24/39	185/208^d^	10/35^b^ ^,^ ^c^	**.008** ^e^
Smoking status, n (%)					.427^e^
Current/former smoker	65 (9.2)	5 (7.9)	25 (6.4)	4 (8.9)	
Never smoker	642 (91.8)	58 (92.1)	368 (93.6)	41 (91.1)	
Symptoms, n (%)					
Cough	420 (59.4)	32 (50.8)	207 (52.7)	27 (60.0)	.122^e^
Sputum	430 (60.8)	37 (58.7)	232 (59.0)	32 (81.1)	.460^e^
Shortness of breath	305 (43.1)	23 (36.5)	165 (42.0)	27 (60.0)	.087^e^

Data are presented as absolute numbers (percentages) in case of frequencies, median values and quartiles in case of continuous parameters.

^a^ *p* < 0.05, underweight group versus the remaining groups;

^b^ *p* < 0.05, normal weight group versus the remaining groups;

^c^ *p* < 0.05, overweight group versus the remaining groups;

^d^ *p* < 0.05, obesity group versus the remaining groups.

^e^ *p*-value is calculated using χ2 test. *p-value is calculated using Kruskal–Wallis tests.

Significant *p*-values indicated in bold text. BMI, body mass index.

### BMI categories difference in spirometry indices

Within the COPD cohort, the normal weight group was used as the reference, underweight group had lower pre-and post FEV_1_ (L, %), pre- and post FVC (L, %), and pre-and post-FEV_1/_FVC (absolute value, %) (all *p* < 0.0001). Overweight and obesity groups had higher pre- and post FEV_1_ (L, %) (all *p* < 0.05), and pre-and post-FEV_1/_FVC (absolute value, %) (all *p* < 0.0001). However, pre-and post FVC (L, %) showed no significant differences (Table 3; Table 4; Table 5).

**TABLE 3:** BMI category differences in FEV_1_ in patients with COPD.

Parameters	Normal weight (N = 2496)	Underweight (N = 675)		Overweight (N = 646		Obesity (N = 74)	
	Median (quartiles)	Median (quartiles)	*P* * value	Median (quartiles)	P* value	Median (quartiles)	P* value
Pre-FEV1 (L)	1.1 (0.8, 1.6)	0.8 (0.6, 1.1)	**< .0001**	1.3 (0.9, 1.8)	**< .0001**	1.4 (0.9, 1.9)	**.035**
Pre-FEV1 (%)	42.5 (30.1,60.5)	31.6 (23.2, 42.8)	**< .0001**	51.9 (38.0, 66.6)	**< .0001**	56.3 (37.5, 70.5)	**.002**
Post-FEV1 (L)	1.2 (0.9, 1.7)	0.9 (0.7, 1.2)	**< .0001**	1.4 (1.0, 1.9)	**< .0001**	1.5 (1.0, 2.0)	**.021**
Post-FEV1 (%)	46.6 (33.4, 65.2)	34.8 (25.5,45.2)	**< .0001**	55.6 (41.3, 72.0)	**< .0001**	60.8 (42.3,76.4)	**.003**

P*: compared with Normal weight group. Significant *p*-values indicated in bold text. *p*-value is calculated using Kruskal–Wallis tests.

BMI, body mass index; FEV_1_, forced expiratory volume in the first second; %, %predicted.

Pre, spirometry measurements before bronchodilator administration.

Post, spirometry measurements after bronchodilator administration.

**TABLE 4:** BMI category differences in FVC in patients with COPD.

Parameters	Normal weight (N = 2496)	Underweight (N = 675)		Overweight (N = 646		Obesity (N = 74)	
	Median (quartiles)	Median (quartiles)	P* value	Median (quartiles)	P* value	Median (quartiles)	P* value
Pre-FVC (L)	2.6 (2.1, 3.2)	2.2 (1.8, 2.7)	**< .0001**	2.7 (1.9, 3.2)	.115	2.5 (1.9, 3.0)	0.087
Pre-FVC (%)	78.6 (65.0, 92.1)	67.2 (54.7, 80.2)	**< .0001**	79.4 (67.6, 93.6)	.315	75.5 (58.3, 90.5)	.166
Post-FVC (L)	2.8 (2.3, 3.4)	2.4 (1.9, 2.8)	**< .0001**	2.8 (2.3, 3.4)	.326	2.5 (2.1, 3.2)	.409
Post-FVC(%)	83.8 (69.8, 96.5)	70.8 (57.5, 83.0)	**< .0001**	83.9 (71.0, 97.3)	.300	80.9 (60.9,94.8)	.528

P*: compared with Normal weight group. Significant *p*-values indicated in bold text. *p*-value is calculated using Kruskal–Wallis tests.

BMI, body mass index; FVC, forced vital capacity; %, %predicted.

**TABLE 5:** BMI category differences in FEV_1_/FVC in patients with COPD.

Parameters	Normal weight (N = 2496)	Underweight (N = 675)		Overweight (N = 646		Obesity (N = 74)	
	Median (quartiles)	Median (quartiles)	*P* * value	Median (quartiles)	*P* * value	Median (quartiles)	*P* * value
Pre-FEV1/FVC (absolute)	0.43 (0.35, 0.55)	0.37 (0.31, 4.73)	**< .0001**	0.51 (0.41, 0.62)	**< .0001**	0.59 (0.49, 0.69)	**< .0001**
Pre-FEV1/FVC (%)	52.6 (42.2, 65.8)	44.3 (37.6, 56.4)	**< .0001**	61.6 (50.0, 74.5)	**< .0001**	70.2 (59.0, 84.2)	**< .0001**
Post-FEV1/FVC (absolute)	0.44 (0.35, 0.56)	0.38 (0.31, 0.48)	**< .0001**	0.53 (0.41, 0.63)	**< .0001**	0.60 (0.51, 0.70)	**< .0001**
Post-FEV1/FVC (%)	53.3 (38.0, 58.5)	45.7 (38.0, 58.5)	**< .0001**	63.6 (50.0, 77.1)	**< .0001**	72.5 (60.6, 87.6)	**< .0001**

P*: compared with Normal weight group. Significant *p*-values indicated in bold text. *p*-value is calculated using Kruskal–Wallis tests.

Abbreviations see Table 3 and Table 4.

Table 6, Table 7, and Table 8 showed results of patients with asthma that used normal weight group as the reference. Underweight group had lower pre-and post FEV_1_ (L, %) (all *p* < 0 .05), pre-and post FVC (L, %) all *p* < 0.05), pre-FEV_1/_FVC (absolute value) (*p* = 0.036), pre-and post- FEV_1/_FVC (%) (*p* = 0.035 and *p* = 0.010, respectively). Overweight and obesity groups had higher pre-FEV_1/_FVC (absolute value, %) (*p* = 0.026, *p* = 0.010, *p* < 0.0001, *p* = 0.010, and *p* = 0.004, respectively). Obesity group showed higher post-FEV_1/_FVC (absolute value, %) (*p* = 0.001 and *p* = 0.030, respectively). What’s more, obesity group had lower pre-and post FVC (L) (*p* = 0.022 and *p* = 0.006, respectively), and overweight group had lower pre-FVC% (*p* = 0.003). However, overweight and obesity groups had no significant differences in pre-and post-FEV1 (L, %) compared with the normal weight group.

**TABLE 6:** BMI category differences in FEV_1_ in patients with asthma.

Parameters	Normal weight (N = 707)	Underweight (N = 63)		Overweight (N = 393		Obesity (N = 45)	
	Median (quartiles)	Median (quartiles)	P* value	Median (quartiles)	P* value	Median (quartiles)	P* value
Pre-FEV1 (L)	1.4 (1.0, 1.9)	1.1 (0.6, 1.4)	**< .0001**	1.4 (1.1, 1.8)	.741	1.3 (1.0, 1.6)	.538
Pre-FEV1 (%)	60.0 (44.4,73.6)	49.7 (31.8, 64.4)	**< .007**	59.3 (46.0,70.2)	.984	60.5 (52.9, 74.6)	.111
Post-FEV1 (L)	1.6 (1.2, 2.0)	1.2 (0.8, 1.6)	**< .0001**	1.6 (1.2, 2.0)	.823	1.5 (1.2, 2.1)	.789
Post-FEV1 (%)	67.1 (52.9, 80.7)	57.8 (39.2, 69.4)	**.013**	67.4 (53.1,79.0)	.809	72.0 (59.2,82.7)	.192

P*: compared with Normal weight group. Significant *p*-values indicated in bold text. *p*-value is calculated using Kruskal–Wallis tests.

Data are presented as median values and quartiles. Abbreviations see Table 3.

**TABLE 7:** BMI category differences in FVC in patients with asthma.

Parameters	Normal weight N = 707	Underweight N = 63		Overweight N = 393		Obesity N = 45	
	Median (quartiles)	Median (quartiles)	P* value	Median (quartiles)	P* value	Median (quartiles)	P* value
Pre-FVC (L)	2.5 (2.0, 3.1)	2.1 (1.6, 2.5)	**< .0001**	2.5 (2.0, 3.1)	.118	2.1 (1.8, 2.7)	**.022**
Pre- FVC%	86.2 (73.1, 99.7)	81.6 (66.7, 91.4)	**.013**	82.1 (72.0, 93.5)	**.003**	81.4 (70.0, 91.4)	.162
Post- FVC (L)	2.7 (2.2, 3.3)	2.1 (1.8, 2.8)	.139<. **< .0001**	2.7 (2.1, 2,8)	.598	2.2 (1.9, 2.8)	**.006**
Post-FVC%	92.4 (79.0, 104.6)	85.7 (70.5, 96.1)	**< .0001**	89.6 (78.0, 99.9)	.150	86.4 (77.0, 100.1)	.145

P*: compared with Normal weight group. Significant *p*-values indicated in bold text. *p*-value is calculated using Kruskal–Wallis tests.

Data are presented as median values and quartiles. Abbreviations see Table 4.

**TABLE 8:** BMI category differences in FEV_1_/FVC in patients with asthma.

Parameters	Normal weight N = 707	Underweight N = 63		Overweight N = 393		Obesity N = 45	
	Median (quartiles)	Median (quartiles)	P* value	Median (quartiles)	P* value	Median (quartiles)	P* value
Pre-FEV1/FVC (absolute value)	0.56 (0.47, 0.65)	0.51 (0.39, 5.98)	**.036**	0.58 (0.50, 0.66)	**.026**	0.63 (0.55, 0.71)	**<.0001**
Pre-FEV1/FVC%	67.1 (55.8, 78.0)	62.6 (47.3, 72.7)	**.035**	70.3 (60.8, 79.8)	**.010**	74.5 (64.8, 80.8)	**.004**
Post-FEV1/FVC (absolute value)	0.59 (0.49, 0.68)	0.54 (0.40, 0.66)	**.161**	0.62 (0.52, 0.70)	**.093**	0.66 (0.58, 0.73)	**.001**
Post-FEV1/FVC%	71.6 (59.9, 81.2)	63.6 (48.1, 78.6)	**.010**	74.7 (63.2, 84.1)	**.161**	78.3 (69.0, 85.9)	**.030**

P*: compared with Normal weight group. Significant *p*-values indicated in bold text. *p*-value is calculated using Kruskal–Wallis tests. Abbreviations see Table 3 and Table 4.

## Discussion

In the present study, the association of BMI categories of spirometry indices in patients with COPD or asthma were analyzed, respectively. In COPD, the prevalence of underweight was much higher than in obese patients. Underweight could worsen the FEV_1_%, FVC%, as well as the FEV_1_/FVC (absolute value, %), conversely overweight and obesity could make FEV_1_% and FEV_1_/FVC (absolute value, %) much better in COPD patients. On contrary, patients with asthma showed higher prevalence of overweight and obesity than underweight. Within the cohort of asthma, underweight also decreased FEV_1_%, FVC%, and FEV_1_/FVC (absolute value, %), but overweight and obesity did not affect spirometry indices except FEV_1_/FVC.

In patients with COPD, the proportion of the underweight category is near 20%, moreover, this category was older than the normal weight category. According to a report from World Health Organization, undernutrition is among the key driver of other respiratory diseases, like tuberculosis (World Health Organization, 2022). It serves as a reminder that undernutrition in older COPD patients should be more closely monitored by patients and physicians. Conversely, patients with asthma had a higher prevalence of overweight and obesity. Obesity is regarded as a health epidemic worldwide (Afshin et al., 2017). In asthma, more attention should be paid to the effect of higher BMI categories on pulmonary function.

The association between BMI and lung function in respiratory diseases has been previously examined. Schaeffer et al. (Schaeffer et al., 2022) used linear regression to assess the association of BMI with lung function in interstitial lung disease. They found that total lung capacity and residual volume% were worse in patients with overweight and obese BMI, and the obesity was associated with a lower FVC%. What’s more, the underweight BMI category was also associated with a higher residual volume%. Lee et al. (Lee et al., 2022) explored the risk of impaired lung function among obese individuals without metabolic abnormalities, subjects with metabolically-healthy obesity had the highest FEV_1_% and FVC% but the lowest FEV_1_/FVC ratio compared with non-obese and metabolically unhealthy obese subjects. Another study included 72 men with COPD also showed that BMI was positively correlated with FEV_1_ (r = 0.275, *p* = 0.020) (Sami et al., 2018). Although there are some similarities to the results of previous studies, our findings have differences. We assessed both pre-BDTs and post-BDTs indices, especially the post-FEV_1_/FVC ratio< 0.7 is the most crucial parameter for diagnosing COPD, so it is important to assess the association of BMI with this parameter. In addition, our research found no significant difference in FEV_1_ and FVC but a higher FEV_1_/FVC ratio in obese asthma patients, which is inconsistent with Lee et al. (Lee et al., 2022). The lower trend of FVC and subtle change of FEV_1_ may be the reason for the higher FEV_1_/FVC.

The present study investigated the association of BMI categories with spirometric indices based on patients with moderate to severe ventilatory dysfunction in COPD and asthma. It demonstrates the harm of the underweight BMI category for pulmonary function in both COPD and asthma, which may be explained by nutrient deficiencies. Our findings on gained residual volume in underweight individuals are consistent with a previous study in interstitial lung disease (Schaeffer et al., 2022). In overweight and obesity groups, the higher spirometry indices in COPD were observed. It may due to restriction by the loading of obesity reduced hyperinflation from the balance of obstructive respiratory disease (Ora et al., 2009; Choe et al., 2018). However, excess fat not only promotes the secretion of a range of inflammatory factors, but fat accumulation can also cause internal organs to be squeezed, which affects the breathing of the lung function (Choe et al., 2018). Hence, the relationship between obesity and lung function in COPD patients remains to be further clarified. In contrast, the worse trend of FVC% were found in higher BMI categories in asthma. It is possible due to excess weight exacerbates the restrictive defect of asthma. A previous study also demonstrated reduced lung volumes in obese subjects without lung disease (Jones and Nzekwu, 2006).

Despite the study having explored the association of BMI with spirometry indices, it had some limitations. Firstly, the data we used was all acquired from BDTs, since most of the time BDT was completed when FEV_1_% < 70%, subjects with normal or mild severity ventilatory dysfunction would be mostly excluded. As a result, we could not know the effect of BMI on these populations. In the future, we should include these subjects to generalize the scope of the study. Secondly, the BDTs records we used were all from the Chinese population. Considering that race is one of the critical impactors of normal spirometry values, it is possible that BMI categories have different effects on other ethnicities. Additionally, we only evaluated COPD and asthma, other common chronic obstructive respiratory diseases, such as bronchiectasis, cystic fibrosis, etc., were not studied.

## Conclusion

To conclude, the lowest BMI has a negative impact on spirometry indices in both COPD and asthma. Increasing BMI may positively affect pulmonary function in COPD. Pulmonologists and physicians should pay attention to these effects to better diagnose and manage patients. Maintaining a suitable BMI is important for patients with asthma or COPD. Weight maintenance should be embedded in the management of these illnesses.

## Data Availability

The raw data supporting the conclusion of this article will be made available by the authors, without undue reservation.

## Abbreviations

BDT, bronchodilator test; BMI, body mass index; COPD, chronic obstructive pulmonary disease; FEV_1_, forced expiratory volume in 1s; FVC, forced vital capacity; %, %predicted.
